# Antagonistic Effects of Light Pollution and Warming on Habitat‐Forming Seaweeds

**DOI:** 10.1002/ece3.70420

**Published:** 2024-10-16

**Authors:** Amelia Caley, Ezequiel M. Marzinelli, Maria Byrne, Mariana Mayer‐Pinto

**Affiliations:** ^1^ Centre for Marine Science and Innovation, Evolution & Ecology Research Centre, School of Biological, Earth and Environmental Science University of New South Wales Sydney New South Wales Australia; ^2^ School of Life and Environmental Sciences The University of Sydney Sydney New South Wales Australia

**Keywords:** kelp, light pollution, macroalgae, multiple stressor

## Abstract

Artificial Light at Night (ALAN) is an emerging global stressor that is likely to interact with other stressors such as warming, affecting habitat‐forming species and ecological functions. Seaweeds are dominant habitat‐forming species in temperate marine ecosystems, where they support primary productivity and diverse ecological communities. Warming is a major stressor affecting seaweed forests, but effects of ALAN on seaweeds are largely unknown. We manipulated ALAN (0 lx vs. 25 lx at night) and temperature (ambient vs. +1.54°C warming) to test their independent and interactive effects on the survival, growth (biomass, total‐, blade‐ and stipe‐length) and function (photosynthesis, primary productivity and respiration) on the juveniles of two habitat‐forming seaweeds, the kelp *Ecklonia radiata* and the fucoid *Sargassum* sp. Warming significantly increased *Ecklonia* mortality; however, ALAN did not affect mortality. ALAN had positive effects on *Ecklonia* biomass, total and blade growth rates and gross primary productivity; however, warming largely counterbalanced these effects. We found no significant effects of warming or ALAN on *Ecklonia* photosynthetic yield, stipe length, net primary productivity or respiration rates. We found no effects of ALAN or warming on *Sargassum* for any of the measured variables. *Synthesis.* Our findings indicate that ALAN can have positive effects on seaweed growth and functioning, but such effects are likely species‐specific and can be counterbalanced by warming, suggesting an antagonistic interaction between these global stressors. These findings can help us to predict and manage the effects of these stressors on seaweeds, which underpin coastal biodiversity.

## Graphical abstract

Artificial Light at Night (ALAN) is an emerging global stressor that is likely to interact with ocean warming to affect ecological functions. Seaweeds are dominant habitat‐forming species in temperate marine ecosystems, where they support primary productivity and diverse ecological communities. We manipulated ALAN and warming to look at their independent and interactive effects on two species of seaweeds. ALAN increased some seaweed growth metrics; however, these effects were species‐specific and were largely counterbalanced by warming.

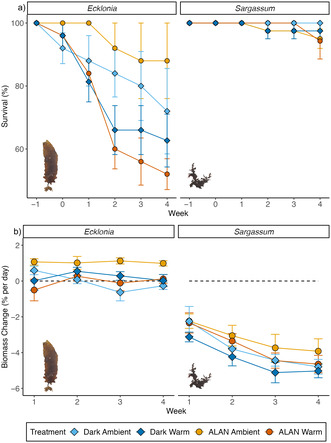

## 1 Introduction

Artificial Light at Night (ALAN) is an emerging global stressor in terrestrial and marine ecosystems (Davies et al. 2014; Falchi et al. 2016). By disrupting natural daylight cycles and masking moonlight, ALAN can impact the health and functioning of a wide range of organisms through physiological and behavioural changes (Gaston et al. 2013). Of particular concern are the effects of ALAN on habitat‐forming species, which could have flow‐on consequences for communities and ecosystem functioning (Fobert et al. 2023; Sanders et al. 2023). Primary producers play a critical role in habitat formation, food provision and nutrient cycling across terrestrial, aquatic and marine biomes (Jackson et al. 2001; Teagle et al. 2017). As light is used by primary producers as a source of both energy and information, altered primary production has been identified as a major pathway by which ALAN can cause cascading ecosystem effects (Fobert et al. 2023; Sanders et al. 2023). While the effects of ALAN have been studied in a range of terrestrial (Sanders et al. 2018; Hey et al. 2020; Murphy et al. 2022) and freshwater primary producers (Poulin et al. 2013; Hölker et al. 2015; Grubisic 2018; Mondy et al. 2021), the effects of ALAN on marine primary producers are largely unknown.

Shallow coastal habitats are among the Earth's most biologically diverse and productive habitats (Tittensor et al. 2010). However, these habitats are also vulnerable to ALAN, with 22% of global coastlines (Davies et al. 2014) and 1.6 million km^2^ of coastal seas at 10 m depth exposed to biologically significant levels of light pollution (Smyth et al. 2021). Seaweeds are key marine primary producers and are the dominant habitat‐formers that underpin biodiversity and productivity in temperate coastal reefs (Teagle et al. 2017; Pessarrodona et al. 2022; Cotas et al. 2023). However, studies investigating the effects of ALAN on marine producers are not only few but have been mainly limited to seagrass (Dalle Carbonare et al. 2023), biofilms (Maggi and Benedetti‐Cecchi 2018; Maggi, Bertocci, and Benedetti‐Cecchi 2020; Maggi et al. 2020) and phytoplankton (Diamantopoulou et al. 2021). One previous study exposed kelp to ALAN and warming, with the focus on sea urchin consumption of kelps (Caley et al. 2024). However, in this study, with respect to the seaweeds, only C:N ratio and photosynthetic yield were measured. ALAN can affect photosynthetic organisms through many different pathways, including increasing available light for photosynthesis at high intensities (Briggs 2006; Raven and Cockell 2006; Maggi and Benedetti‐Cecchi 2018), or altering biomass allocation (Murphy et al. 2022), light‐signalling processes (Dalle Carbonare et al. 2023) or other light‐mediated traits (Segrestin et al. 2021). In some terrestrial plants, freshwater photoautotrophs, marine biofilms and phytoplankton, ALAN can increase growth and biomass (Hölker et al. 2015; Maggi and Benedetti‐Cecchi 2018; Sanders et al. 2018; Diamantopoulou et al. 2021; Murphy et al. 2022), although effects may be counterbalanced by increased herbivory under ALAN (Maggi and Benedetti‐Cecchi 2018; Mondy et al. 2021; Caley et al. 2024). In other primary producers, however, such as aquatic periphyton and seagrass, ALAN can negatively impact growth and biomass (Grubisic 2018; Dalle Carbonare et al. 2023). Therefore, ALAN is expected to affect the growth and productivity of seaweeds, but the direction (e.g., positive or negative) and magnitude of effects are likely to be species‐specific. Although previous research has shown that seaweeds, like plants, are affected by changing light intensity and spectra (e.g., white, red, green and blue), these studies have focused on the effects of artificial light during daytime hours, rather than the effects of artificial light at night (e.g., Huang et al. 2021; Torres et al. 2023). With the global extent of light pollution steadily increasing (Kyba et al. 2017), it is necessary to specifically understand how nighttime light pollution might affect these ecologically important habitat‐forming species.

Globally, seaweed forests are threatened by a wide range of stressors. In particular, ocean warming is rapidly altering the distribution and composition of these underwater forests (Wernberg et al. 2011; Krumhansl et al. 2024). The effects of ocean warming on seaweeds are species‐specific and include reduced survival, photosynthetic efficiency and growth, as well as increased C:N ratio, bleaching and palatability (Phelps, Boyce, and Huggett 2017; Straub et al. 2022; Castro et al. 2024). Critically, due to its widespread extent (Davies et al. 2014, 2020; Smyth et al. 2021), ALAN is expected to increasingly co‐occur with ocean warming. Yet, we know very little about the combined effects of these stressors on coastal habitats. ALAN may, for example, have positive effects on seaweed growth, survival and productivity, which could offset the predicted negative effects of warming. Alternatively, ALAN may negatively affect seaweeds, exacerbating impacts from warming, i.e., synergistic effects. Understanding how these stressors interact is important for the conservation and management of these key organisms.

Underwater forests often comprise multiple habitat‐forming seaweed species, including kelp (Laminariales) and fucoid species, which have distinct ecological functions and responses to stressors (Coleman and Wernberg 2017; Teagle et al. 2017; Straub et al. 2022; Castro et al. 2024). In Australia, the kelp *Ecklonia radiata* is found on temperate and subtropical subtidal rocky reefs along the entire southern edge of the continent, known as the Great Southern Reef (GSR) (Wernberg et al. 2019). The GSR supports high biodiversity, including endemic and commercially significant species (Kerswell 2006; Bennett et al. 2016) and contributes $10 billion per year to fisheries, tourism and recreation industries (Bennett et al. 2016). The fucoid *Sargassum* is also widely distributed in the GSR and is the most speciose genus of macroalgae in this region, with high levels of endemism (Millar and Kraft 1994; Coleman and Wernberg 2017). *Sargassum* supports distinct invertebrate communities with similar or higher diversity and abundance as *E. radiata* (Marzinelli et al. 2016; Coleman and Wernberg 2017). As *Sargassum* is semi‐perennial (canopy is annual), it also provides an important source of nutrient subsidies to other systems (Bishop, Coleman, and Kelaher 2010; Coleman and Wernberg 2017). Impacts of light pollution and warming on these seaweed species are therefore expected to have profound impacts on temperate reef systems.

In this first study of the interactive effects of ALAN and warming on seaweeds, we exposed juvenile *Ecklonia radiata* (hereafter *Ecklonia*) and *Sargassum* spp. (hereafter *Sargassum*) to these stressors, by manipulating ALAN (0 lx vs. 25 lx) and temperature (ambient vs. + 1.54°C) under laboratory conditions for 4 weeks. We assessed the effects of these stressors on the survival, growth, photosynthetic efficiency, respiration rates and net and gross primary productivity of both species. We hypothesised that, on its own, ALAN would increase survival, growth, photosynthetic yield, net primary productivity (NPP) and gross primary productivity (GPP) but decrease respiration rates (R) in both species. Conversely, we hypothesised that warming would have opposite effects, decreasing most variables measured in both species, except for respiration rates, which we predicted would increase in response to stress. We also predicted that warming would have greater impacts on *Ecklonia* than on *Sargassum*, based on previous studies (Xiao et al. 2015; Straub et al. 2022; Castro et al. 2024). In combination, we predicted negative, synergistic impacts of ALAN and warming on the seaweeds.

## 2 Materials and Methods

### 2.1 Collections and Treatments

We collected 102 *Ecklonia radiata* juveniles and 151 *Sargassum* juveniles from a naturally dark (i.e., low ALAN exposure) rocky shore in Chowder Bay, Sydney Harbour, Australia (33°50′26.1″ S, 151°15′09.6″ E) in September 2023 (austral spring) and transported them to the nearby (~300 m away) Sydney Institute of Marine Science (SIMS). Seaweed was collected under an s37 Research Permit (no. FP23/6) issued by DPI, NSW. *Ecklonia* juveniles (mean length 144.36 mm ± 4.27 SE) were defined as stage I early sporophytes with a single main blade and no lateral laminae (Figure S1) (Kirkman 1984). Juvenile *Sargassum* sp. were defined as small *Sargassum* sp. < 200 mm (mean length 81.74 mm ± 2.45 SE) (Straub et al. 2022) (Figure S1). Because of their high morphological and species diversity, *Sargassum* is difficult to distinguish to the species level without detailed microscopic examination (Coleman and Wernberg 2017), and so we just use the genus name here. We used juveniles as they are a foundational life stage for seaweed populations, making their vulnerability to stressors important to understand because of implications for recruitment and recovery from disturbances. Their smaller size also allowed us to incorporate whole individuals into our mesocosms.

Individual seaweeds were randomly distributed across tanks within 4 h of collection. Each individual was carefully attached to small rocks (30–50 mm decorative pebbles) using elastic bands (Figure S2) (Gonzalez et al. 2024). *Ecklonia* tanks contained five to six individuals, while *Sargassum* tanks contained seven to eight individuals. Tanks used were 45 L, so differences in the number of individuals per tank were assumed to have negligible effects. Tanks were supplied with a constant flowthrough supply of seawater drawn from 8 m depth in Chowder Bay, the location where the seaweeds were collected. Seawater was filtered to 100 μm and supplied to tanks at a rate of 5 L/min. Tanks were also cleaned twice a week and supplied with moderate aeration, using air pumped through air stones. Seaweeds were acclimatised for 1 week at ambient seawater temperatures (Straub et al. 2022). All tanks were lit at ~3000 lx during the day with cool white light (6500 K, 150 cm LED tubes from MakeMyLED). This is within the range of daily lux levels naturally experienced by kelp (Bearham, Vanderklift, and Gunson 2013) and is equivalent to natural daylight levels measured under cloudy conditions at 1 m depth in Chowder Bay, using an underwater lux meter (SpectroSense2; Skye, Wales). After acclimation, tanks were randomly allocated to one of four treatments: ALAN/warm, ALAN/ambient, dark/warm or dark/ambient (*n* = 5 tanks). Dark tanks were assigned to a 12 h light (~3000 lx) and 12 h dark (0 lx) cycle. ALAN treatments had 12 h of light (~3000 lx) during the day and 12 h of dim light during the night (25 lx). Although these light levels are higher than what has been detected on some shores in Sydney Harbour (Trethewy, Mayer‐Pinto, and Dafforn 2023), they are within the range of levels that can occur directly under light sources along highly urbanised shorelines (Bauer et al. 2022) or near ports and/or other infrastructure (Bolton et al. 2017). While these levels may not be commonly found in situ, they allow us to understand the mechanisms and effects of ALAN (Marangoni et al. 2022), which is increasing in intensity and extent globally (Kyba et al. 2017). Warm treatment tanks were heated to 1.5°C above ambient seawater conditions using 300 watt bar heaters (Thermocontrol 300; EHEIM, Germany), while the rest were unmanipulated (ambient seawater conditions). Temperatures in both ambient and warm treatments were allowed to fluctuate to reflect natural temperature variability (Figure S3). This was achieved as all tanks received a continuous flowthrough of ambient seawater from 8 m depth, but warm treatment tanks were heated with bar heaters, which allowed temperatures to fluctuate similarly in both treatments. HOBO Pendant Loggers (Onset, USA) measured temperature at 30‐min intervals for the entire experiment. Mean temperatures were 18.92 ± 0.002°C in ambient treatments and 20.46 ± 0.003°C in warm treatments over the experiment (mean difference of +1.54°C; Figure S3). The mean daily temperature ranged between 18.15°C and 19.58°C for ambient treatments and 19.11°C and 21.43°C for warm treatments (Figure S3).

### 2.2 Survival and Biomass Change

Within each tank, three individual seaweeds were randomly tagged for measurement. A two‐factor ANOVA confirmed there was no significant difference in the size of tagged individuals among treatments at the start of the experiment (Table S1, *p* > 0.5 for all variables). Survival was recorded weekly as % of survival of all individuals within a tank (tagged and untagged). Seaweeds were characterised as ‘dead’ when the specimen lost its structural integrity, i.e., the stipe was fully detached from the blade or the blade tissue was fully degraded (Straub et al. 2022). We also measured biomass, length and photosynthetic efficiency weekly for tagged individuals. Seaweeds were blotted dry and weighed using scales to measure biomass as the total wet weight, to 0.01 g. For *Ecklonia*, we measured total length (base of the stipe to the top of the central blade), blade length (top of the stipe to the top of the central blade) and stipe length (base of the stipe to the base of the blade). For *Sargassum*, the total length was measured as the distance from the base of the stipe (above the holdfast) to the top of the longest blade. We did not measure the blade length and stipe length of *Sargassum* due to its variable morphology. We calculated the daily relative growth rate (‘change’) of biomass, total length, blade length and stipe length as the proportional increase in biomass or length per day, using the following equation:

$$\text{Relative Growth Rate}\;\left(\%{\mathrm{day}}^{-1}\right)=\left[{\left(\frac{W_{t1}}{W_{i}}\right)}^{\frac{1}{t}}-1\right]\times 100$$

We calculated RGR cumulatively for each week, where *W*_i_ = initial wet weight (g), total length, blade length or stipe length (mm) at the start of the experiment, *W*_*t*1_ = wet weight (g), total length, blade length or stipe length (mm) at the respective measurement week and *t* = days passed in treatment, as per Straub et al. (2022). This formula has been determined to be the most accurate for calculating seaweed growth rates (Yong, Yong, and Anton 2013). Change could be positive (due to growth) or negative (due to the loss of tissue).

### 2.3 Seaweed Functioning

Photosynthetic efficiency (maximum quantum yield) was measured weekly using a pulse‐amplitude‐modulated fluorometer (Diving‐PAM; WALZ, Germany). Whole individual seaweeds were wrapped in foils and dark‐acclimated for 10 min prior to measurement (Mayer‐Pinto, Underwood, and Marzinelli 2015). Three replicate measures were taken and averaged for each individual seaweed (on a single blade for *Ecklonia* and on separate blades for *Sargassum*), to improve precision. Respiration rates (R), gross primary productivity (GPP) and net primary productivity (NPP) were measured at the end of the experiment (after 4 weeks) for *n* = 6 individuals, selected randomly, from each treatment and species. To achieve an appropriate replication level, in some cases, two individuals (maximum) were collected from the same tank. Tank was included as a factor in models to account for potential non‐independence (see below). A closed chamber design was used, and oxygen flux was measured under respective temperature and light treatments, to assess the functioning as it would occur under stressor conditions (Hölker et al. 2015; Gonzalez et al. 2024). For each incubation, a seaweed specimen was placed into a clear plastic 2 L container within the tank, containing a miniDOT logger (White and Davoult 2022). Then, the containers were sealed with tight‐fitting lids, ensuring that no air bubbles were present, and left for an hour in light conditions (~ 3000 lx for all treatments). After an hour of light incubation, chamber lids were removed, and the chambers were left open for 15 min. Lids were then replaced to reseal the containers, and ‘dark’ incubations commenced. Incubations of dark treatments were done in complete darkness, while incubations of ALAN treatments were illuminated with artificial light (25 lx). Incubations were done at the same time of day for all replicates and over 2 days for each species (date was included in models to account for this; see below). Dark‐ and light‐dissolved O_2_ flux rates (mg/L/h) were calculated from the linear regressions of dissolved oxygen concentration and time (Mayer‐Pinto et al. 2023). Respiration (dark) and NPP (light) raw rates were standardised by chamber volume and seaweed wet weight (gWW), to give values as O_2_ μmol/gWW/h. GPP was calculated as NPP—R (gross primary productivity in the absence of respiration).

### 2.4 Statistical Analysis

Data were analysed separately for each species and response variable in R (R Core Team 2022). Linear mixed‐effect models were fitted using the ‘lmer’ function from the ‘lme4’ package (Bates et al. 2014). For repeated measures (biomass growth, vertical growth, stipe growth, blade growth and photosynthetic yield), we treated light (two levels, ALAN and dark) and warming (two levels, ambient and warming) as interactive fixed factors, with time as a covariate, and individual replicate (ID) nested in tank as a random factor to account for repeated measures of the same individuals. In all models, time (week) was treated as numeric, as responses were expected to vary linearly through time. For models testing the effects on gross primary productivity (GPP), net primary productivity (NPP) and respiration (R), we had the same factors as above except time (since measurements were only taken once at the end of the experiment), and with date as a random factor, as incubations were done over 2 days per species. Skewed data were cube‐root‐transformed before analysis to meet assumptions, due to negative values. Gaussian distribution was determined to be the most appropriate distribution for all models. Significance was assessed using a likelihood ratio test via the ‘Anova’ function (type II ANOVA) from the ‘car’ package (Fox et al. 2012). Pairwise post hoc tests were performed using emmeans for interactions involving only categorical variables (Lenth 2023). Where significant interactions involved an interaction with time, emtrends was used to test for the difference in slopes between treatments and to test for significant differences between treatments at each week (0, 1, 2, 3 and 4) (Lenth 2023).

## 3 Results

### 3.1 Survival and Biomass Change

**FIGURE 1:**
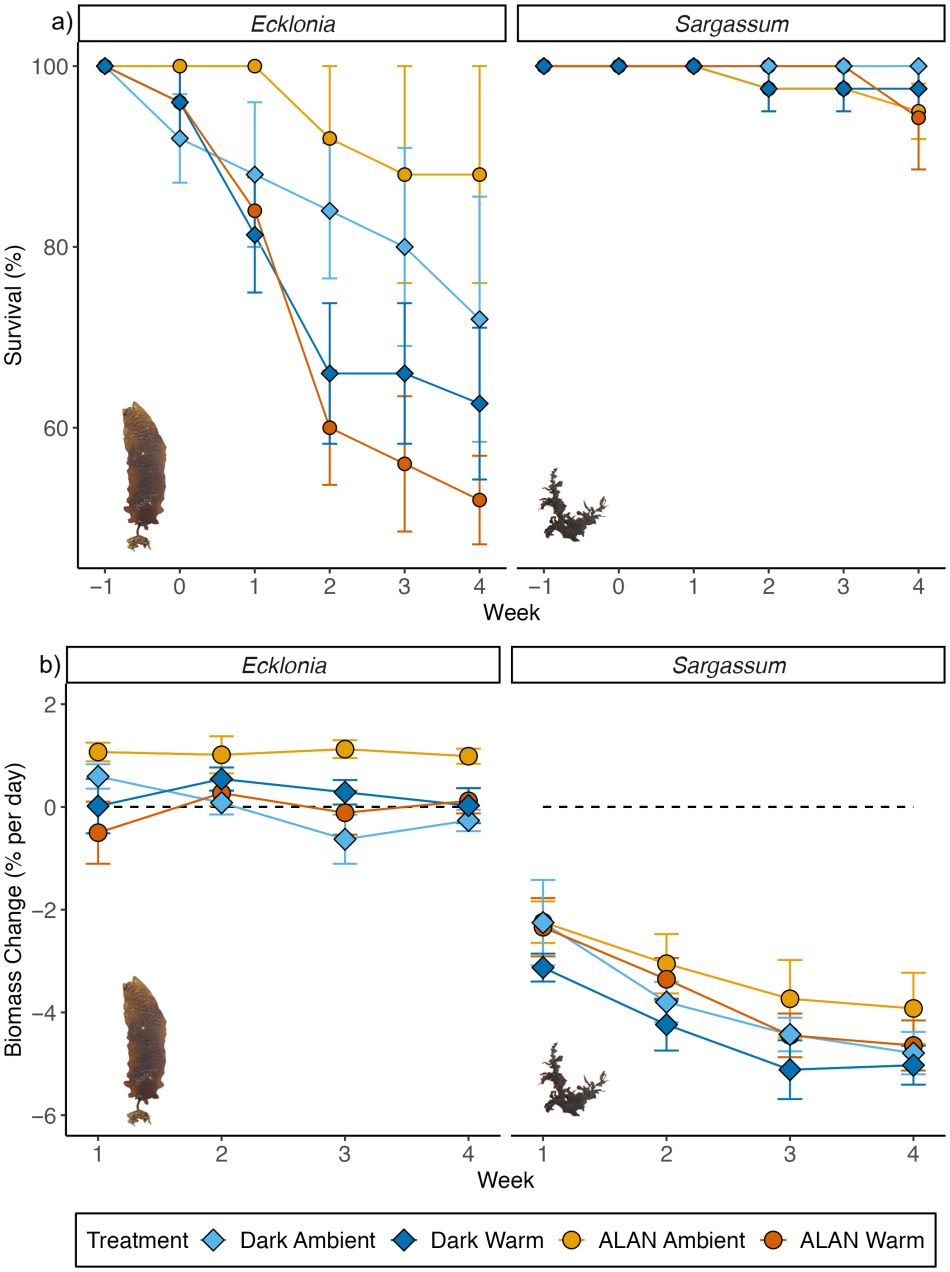
Mean (± SE, *n* = 5 tanks) (a) survival (%) and (b) biomass change (% day^−1^) of *Ecklonia* and *Sargassum* for light and warming treatments. Week −1 in graph (a) represents survival at collection (100%). Baseline measurements were taken at Week 0, after which treatments commenced. Since Week 0 was T0 measurements, biomass change is shown from Week 1. ALAN ambient = yellow diamonds, ALAN warm = orange diamonds, dark ambient = light blue circles and dark warm = dark blue circles. Dotted lines represent 0% biomass change per week, so points above the line indicate a mean increase in biomass per week, while points below the line indicate a mean decrease in biomass per week.

There was a significant interactive effect of warming and time on the survival of *Ecklonia* (Table 1a; Figure 1a). Survival declined over time in both ambient and warm treatments; however, the rate of decline was significantly steeper in warm treatments (Table 1a). From Week 0 to Week 1, survival was not significantly different between treatments; however, from Week 2 onwards, survival was significantly higher in ambient treatments compared to warm treatments (Table 1a). We found no evidence that ALAN affected survival, although survival was the lowest in warm ALAN treatments and highest in ambient ALAN treatments (Table 1a). *Sargassum* survival significantly declined over time across treatments; however, there was no evidence of effects of ALAN or warming, and survival remained close to 100% over the experiment duration (Table 1a; Figure 1a).

**TABLE 1:** Analysis of (a) survival and (b) biomass change of *Ecklonia* and *Sargassum*. Separate models were run for each species using the fixed interacting factors light (two levels: ALAN and dark) and temperature (two levels: ambient and dark), with time as a covariate and individual ID nested in tank as a random factor.

	*Ecklonia*			*Sargassum*		
	Chisq	df	*p*	Chisq	df	*p*
*(a) Survival*						
Light	0.189	1	0.664	0.475	1	0.491
Warming	6.467	1	0.011	0.073	1	0.787
Time	85.687	1	< 0.001	9.560	1	0.002
Light × Warming	1.389	1	0.239	0.978	1	0.323
Light × Time	0.522	1	0.470	2.612	1	0.106
Warming × Time	14.003	1	< 0.001	0.400	1	0.527
Light × Warming × Time	2.280	1	0.131	0.711	1	0.399
	Survival declined significantly over time. Rate of decline was significantly steeper in warm vs. ambient *Week 0*: ambient = warm *Week 1*: ambient = warm *Week 2*: ambient > warm *Week 3*: ambient > warm *Week 4*: ambient > warm			Survival declined significantly over time		
*(b) Biomass change*						
Light	0.115	1	0.734	1.934	1	0.164
Warming	4.213	1	0.040	1.269	1	0.260
Time	18.718	1	< 0.001	68.511	1	< 0.001
Light × Warming	5.465	1	0.019	0.007	1	0.931
Light × Time	3.505	1	0.061	0.033	1	0.857
Warming × Time	0.599	1	0.439	0.001	1	0.971
Light × Warming × Time	5.378	1	0.020	1.018	1	0.313
	Biomass change decreased over time in ambient dark treatments. Differences among treatments per week are detailed below: *Week 1*: Ambient: ALAN = Dark Warm: ALAN = Dark ALAN: Ambient > Warm Dark: Ambient = Warm *Week 2*: Ambient: ALAN = Dark Warm: ALAN = Dark ALAN: Ambient > Warm Dark: Ambient = Warm *Week 3*: Ambient: ALAN > Dark Warm: ALAN = Dark ALAN: Ambient > Warm Dark: Ambient = Warm *Week 4*: Ambient: ALAN > Dark Warm: ALAN = Dark ALAN: Ambient > Warm Dark: Ambient = Warm			Biomass change declined significantly over time		

*Note:* Significant *p*‐values (α < 0.05).

There was a significant interactive effect of light, warming and time on the biomass change of *Ecklonia* (Table 1b; Figure 1b). There was a significant biomass loss over time in ambient dark treatments (~1%) but not in other treatments. In all weeks, biomass growth was significantly higher in ALAN ambient compared to ALAN warm treatments. In weeks 3 and 4, biomass growth was also significantly higher in ALAN ambient compared to dark ambient treatments (Table 1b). There were no significant differences in biomass change in ALAN warm compared to dark warm treatments, or in dark ambient treatments compared to dark warm treatments, in any week (Table 1b). There was no evidence for the effects of ALAN or warming on *Sargassum* biomass change; however, across all treatments, *Sargassum* biomass significantly declined over time, and the rate of change was negative in all weeks (representing a loss of biomass) (Table 1b; Figure 1b).

### 3.2 Relative Total Length, Blade Length and Stipe Length Change

**FIGURE 2:**
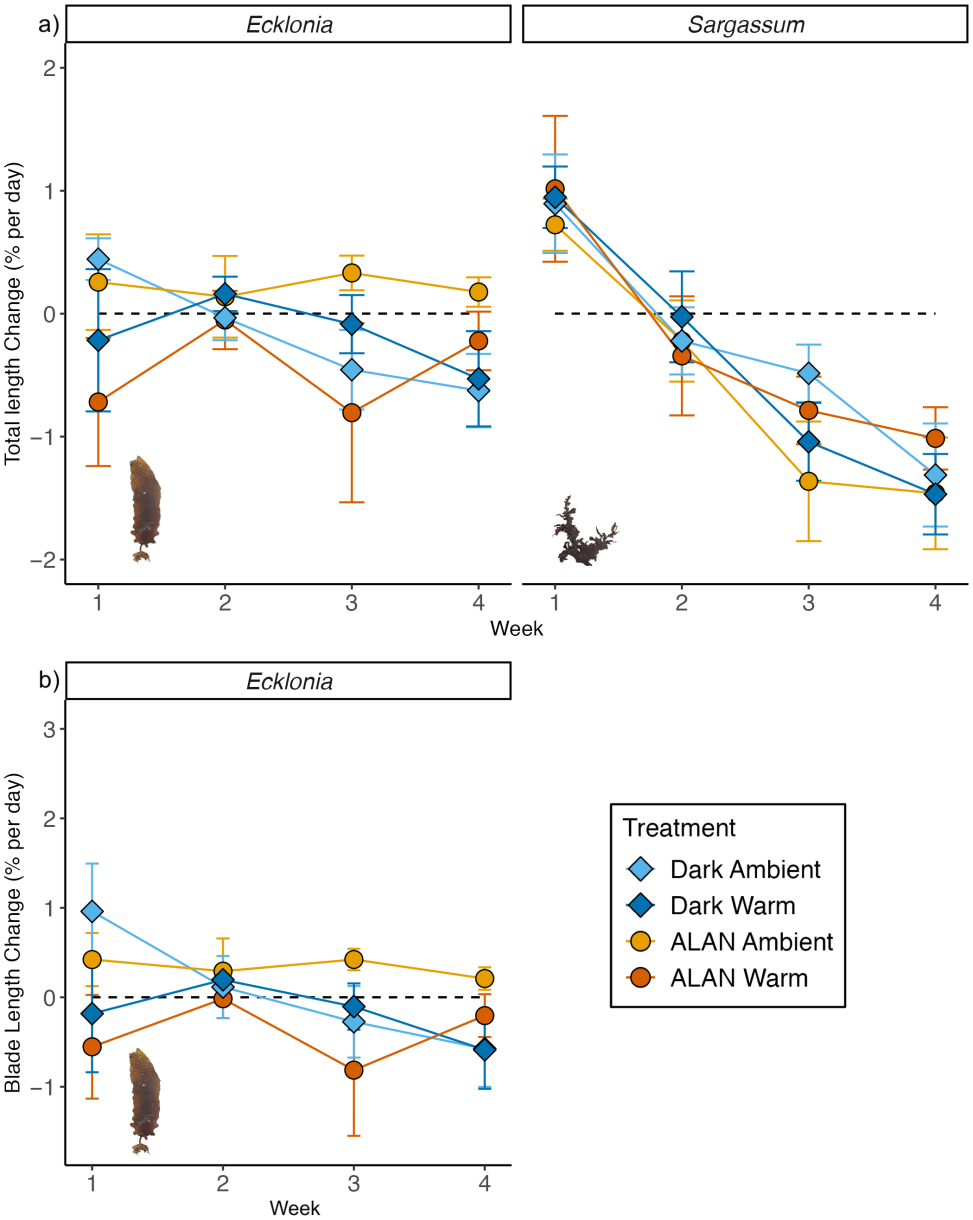
(a) Mean (± SE, *n* = 5 tanks) total length change (% per day) of tagged *Ecklonia* and *Sargassum* individuals and (b) mean (± SE) blade length change of *Ecklonia*. Since Week 0 showed T0 measurements, change rates are shown from Week 1. ALAN ambient = yellow diamonds, ALAN warm = orange diamonds, dark ambient = light blue circles and dark warm = dark blue circles. Dotted lines represent 0% change per week, so points above the line indicate a mean increase in length per week, while points below the line indicate a mean decrease in length per week.

There was a significant interactive effect of light, warming and time on the total and blade length change of *Ecklonia* (Table 2; Figure 2). Change in both the total and blade length of *Ecklonia* significantly decreased in all treatments over time, except in ALAN ambient treatments, where no changes over time were observed (Table 2; Figure 2). Post hoc comparisons showed no significant differences in the total length change among treatments for individual weeks (Table 2a; Figure 2a). Blade growth was greater in ALAN ambient treatments in week 4 compared to dark ambient treatments (Table 2b; Figure 2b). Stipe length change significantly declined over time, but there were no significant effects of light or warming (Table 2b). There were no significant effects of light and warming on the total length change of *Sargassum*; however, the total length significantly decreased over time across all treatments (Table 2a; Figure 2a).

**TABLE 2:** Analysis of (a) *Ecklonia* and *Sargassum* total length change and (b) *Ecklonia* blade and stipe length change per day, using the fixed interacting factors, light (two levels: ALAN and dark) and temperature (two levels: ambient and dark), with time as a covariate and individual ID nested in tank as a random factor.

	*Ecklonia*			*Sargassum*		
	Chisq	df	*p*	Chisq	df	*p*
*(a) Length change*						
Light	0.021	1	0.886	0.121	1	0.727
Warming	0.999	1	0.318	0.119	1	0.730
Time	49.601	1	< 0.001	106.419	1	< 0.001
Light × warming	1.340	1	0.247	0.587	1	0.443
Light × time	5.717	1	0.017	0.108	1	0.742
Warming × time	0.678	1	0.410	0.007	1	0.933
Light × warming × time	6.074	1	0.014	0.813	1	0.367
*Post hoc*	Total length change declined over time in dark ambient, ALAN warm and dark warm treatments but not in ALAN ambient treatments. No significant differences between treatments by week			Total length change significantly declined over time		

	*Ecklonia* blade change			*Ecklonia* Stipe change		
	Chisq	df	*p*	Chisq	df	*p*
*(b) Blade and stipe length change (Ecklonia)*						
Light	0.035	1	0.852	0.282	1	0.595
Warming	2.000	1	0.157	0.002	1	0.967
Time	52.480	1	< 0.001	8.138	1	0.004
Light × warming	1.418	1	0.234	0.252	1	0.616
Light × time	7.445	1	0.006	3.186	1	0.074
Warming × time	2.053	1	0.152	0.009	1	0.923
Light × warming × time	5.677	1	0.017	0.980	1	0.322
Post hoc	Blade length change declined over time in dark ambient, ALAN wand dark warm treatments but not ALAN ambient treatments *Week 1*: No significant differences *Week 2*: No significant differences *Week 3*: No significant differences *Week 4*: Ambient: ALAN > Dark Warm: ALAN = Dark ALAN: Ambient = Warm Dark: Ambient = Warm			Stipe length change significantly declined over time		

*Note:* Significant *p*‐values (α < 0.05).

### 3.3 Seaweed Functioning

**FIGURE 3:**
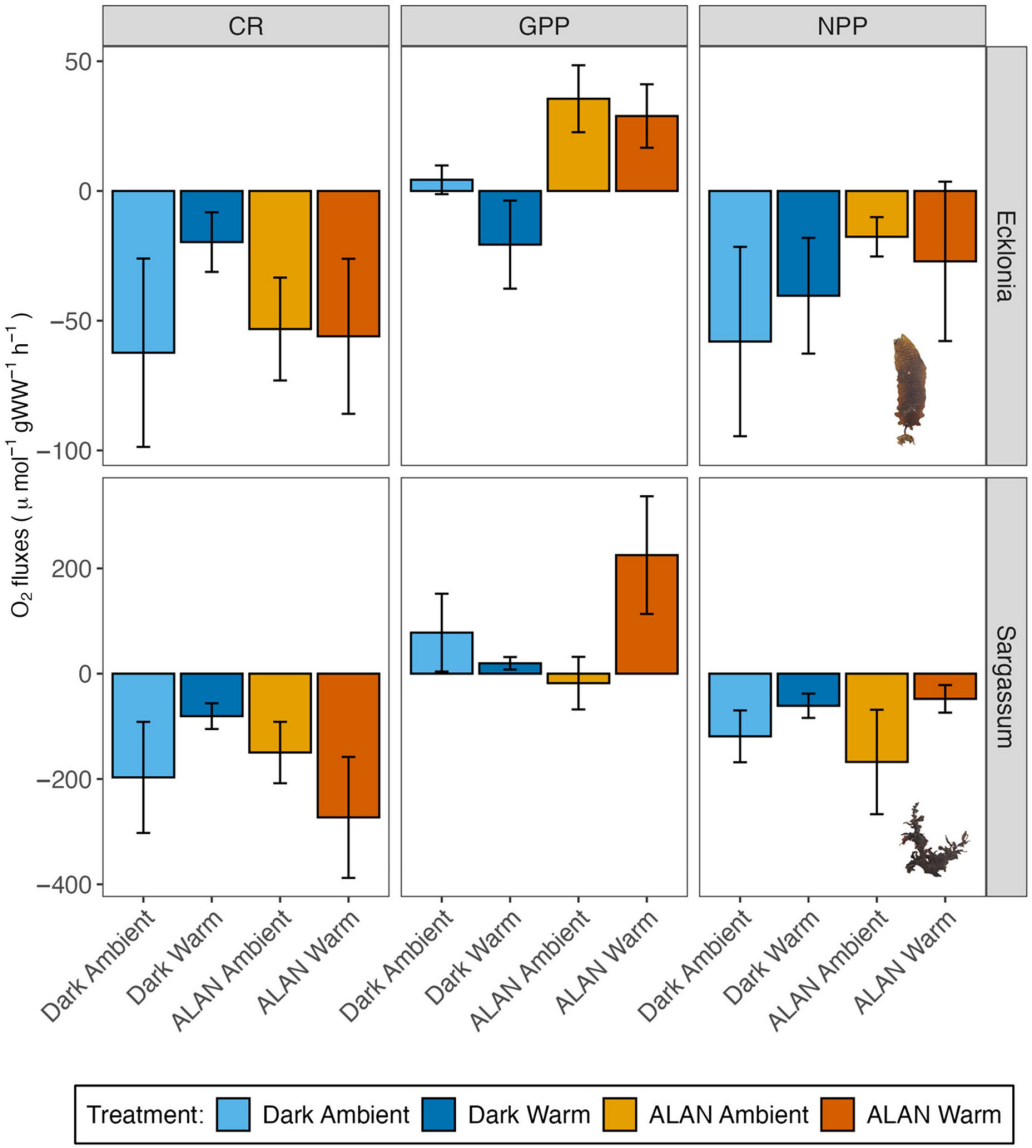
Mean (± SE, *n* = 6 individuals) respiration (R), gross primary productivity (GPP) and net primary productivity (NPP) (O_2_ μmol/gWW/h) of *Ecklonia* (top row) and *Sargassum* (bottom row). ALAN ambient = yellow, ALAN warm = orange, dark ambient = light blue and dark warm = dark blue.

Photosynthetic efficiency of *Ecklonia* or *Sargassum* was not significantly affected by light or warming and did not change over time (Table S2; Figure S4). There were no significant effects of ALAN or warming on respiration or NPP of *Ecklonia* (Table 3; Figure 3). GPP of *Ecklonia* was significantly higher in ALAN treatments compared to dark treatments; however, there was no significant effect of warming (Table 3; Figure 3). We found no significant effects of ALAN or warming on *Sargassum* respiration, NPP or GPP (Table 3; Figure 3).

**TABLE 3:** Analysis of *Ecklonia* and *Sargassum* respiration (R), gross primary productivity (GPP) and net primary productivity (NPP) (O_2_ μmol/gWW/h) using the fixed interacting factors light (two levels: ALAN and dark) and temperature (two levels: ambient and dark), with tank as a random factor.

	Ecklonia			Sargassum		
	Chisq	df	*p*	Chisq	df	*p*
R						
Light	2.224	1	0.136	1.149	1	0.284
Warming	0.392	1	0.531	0.008	1	0.927
Light × warming	0.077	1	0.782	0.522	1	0.470
GPP						
Light	7.134	1	0.008	0.201	1	0.654
Warming	2.407	1	0.121	0.460	1	0.498
Light × warming	0.354	1	0.552	1.380	1	0.240
NPP						
Light	0.008	1	0.927	0.014	1	0.907
Warming	0.043	1	0.836	0.852	1	0.356
Light × warming	0.615	1	0.433	0.729	1	0.393

*Note:* Significant *p*‐values (α < 0.05).

## 4 Discussion

We tested, for the first time, the effects of light pollution and warming on two habitat‐forming seaweeds. Overall, warming significantly decreased *Ecklonia* survival. We found no evidence for the effect of ALAN on survival; however, survival was the lowest in ALAN warm treatments and highest in ALAN ambient treatments. Neither ALAN nor warming affected *Sargassum* survival, as predicted. ALAN, on its own, increased the biomass and blade length of *Ecklonia* as well as gross primary productivity. However, while warming did not independently affect growth, it often counteracted the ‘positive’ effects of ALAN, when in combination. Contrary to our predictions, neither ALAN nor warming affected the *Ecklonia* photosynthetic yield, stipe length or NPP or respiration rates. Similarly, we found no effects of these stressors on any variables measured in *Sargassum*.

Warming significantly decreased the survival of *Ecklonia*, although survival declined over time in all treatments. These results reflect previous studies that showed increased mortality of both adult and juvenile *E. radiata* under similar levels of warming (Phelps, Boyce, and Huggett 2017; Straub et al. 2022). *Ecklonia* is likely to be subject to similar levels of warming as used here in the near future, as south‐eastern Australia is classified as an ocean‐warming hotspot (Hobday and Pecl 2014), and declines of *Ecklonia* due to warming have already been observed in some areas (Wernberg 2021; Young et al. 2023). In contrast, we found no evidence that ALAN affected *Ecklonia* survival. However, survival was lowest in ALAN warm treatments and highest in ALAN ambient treatments, suggesting a synergistic effect. ALAN also significantly affected *Ecklonia* growth.

We found significant interactive effects of ALAN, warming and time on multiple growth metrics for *Ecklonia*. Biomass change captures changes in wet weight due to both vertical and lateral growth or tissue loss, while total length change captures change due to only vertical growth or tissue loss, together providing an overall picture of growth trends. We also measured changes in blade and stipe length over time, since productivity may be allocated differently to tissues under different light and temperature conditions (Blain et al. 2020). Biomass growth was higher in ALAN ambient treatments compared to that in dark ambient treatments across weeks, and in ALAN ambient treatments compared to ALAN warm treatments from week 3 onwards, suggesting a positive independent effect of ALAN on biomass. Results also suggest a positive independent effect of ALAN on the total and blade length of *Ecklonia*, whereas we found no effects of ALAN or warming on the stipe length. This increased growth may have occurred due to increased photosynthetic activity, which can theoretically occur under these levels of ALAN (Raven and Cockell 2006), and has been proposed as the mechanism for increased growth of freshwater photoautotrophs and marine biofilms under ALAN (Hölker et al. 2015; Maggi and Benedetti‐Cecchi 2018). Alternatively, ALAN may have altered metabolic rates, light‐signalling pathways or other traits related to photosynthesis (Hey et al. 2020; Segrestin et al. 2021; Dalle Carbonare et al. 2023). Independently, warming did not impact growth, indicating that lethal effects of warming are more important than sublethal effects for this species, as seen in some other studies (Phelps, Boyce, and Huggett 2017; Straub et al. 2022). However, positive effects of ALAN were only detected in the absence of warming, indicating an antagonistic interaction between warming and ALAN. Critically, some of the observed effects were only detected after 3 or 4 weeks, indicating the importance of conducting studies over longer time spans.

We observed some biomass loss and mortality in control treatments, which may reflect stress caused by weekly measurements and/or tank conditions. However, control treatments had the second highest rate of survival for *Ecklonia* (behind ALAN ambient treatments), and the highest rate of survival for *Sargassum*. Additionally, it is important to note that the biomass loss for *Ecklonia* in control treatments (~1% difference) was mainly driven by two individuals, which died and were removed by week 4. Since ALAN and warming can increase the grazing rates of some herbivores that consume *Ecklonia* (Caley et al. 2024), the positive effects of ALAN on seaweed growth observed here may help to offset this increased grazing pressure but only in the absence of warming. Alternatively, increased growth may eventually lead to higher consumption, as there may be trade‐offs with other traits such as chemical defences (Steinberg 1995) or changes to microbial communities that may influence palatability (Castro et al. 2024).

We found no significant effects of light, warming or time on the maximum photosynthetic yield of *Ecklonia*. This reflects past findings that warming does not impact the photosynthetic yield of *Ecklonia* juveniles (Straub et al. 2022). However, this contrasts with observations that ALAN can alter the photosynthetic yield of some terrestrial, freshwater and marine primary producers (Poulin et al. 2013; Maggi, Bertocci, and Benedetti‐Cecchi 2020; Wei, Li, and Hu 2023). Nevertheless, the increased growth rates observed under ALAN may have been due to the higher overall photosynthetic activity without any increase in the maximum quantum yield, or due to another mechanism. GPP of *Ecklonia* was significantly higher in ALAN treatments compared to dark treatments, irrespective of the warming treatment, supporting the hypothesis of increased photosynthetic activity under ALAN. However, there were no significant differences in respiration rates or NPP between treatments, indicating that this did not translate into higher net productivity. A seawater‐only control chamber was not included in this experiment, so the reported productivity and respiration rates reflect fluxes from both the seaweeds and any organisms (phytoplankton, zooplankton and bacteria) present in seawater. However, all tanks were supplied with the same seawater, which was filtered to 100 μm to remove larger plankton. Additionally, the contribution of these microorganisms to oxygen flux rates was assumed to be negligible compared to that of the seaweeds, as observed in past studies (Gerard 1986; Cheshire et al. 1996) and as the disruption of *Ecklonia* microbial communities has shown no effect on oxygen flux rates (Gonzalez et al. 2024). Nonetheless, future studies should include a water‐only control for each treatment, to more accurately quantify seaweed respiration and productivity.

We found no effects of light and temperature on juvenile *Sargassum*, similar to previous studies (Phelps, Boyce, and Huggett 2017; Straub et al. 2022), which suggests that *Sargassum* is more resilient to these stressors than *Ecklonia*. However, it is important to note that *Sargassum* individuals rapidly lost a large amount of biomass and length across treatments, which could have masked other impacts of warming or ALAN. Similar patterns (i.e., rapid loss of biomass when placed in aquaria) have been observed in previous mesocosm experiments, indicating that *Sargassum* may respond to laboratory conditions by shedding its fronds, which through time can regrow (Straub et al. 2022). Although *Sargassum* can regrow from the holdfast after physical disturbance or loss of biomass (Umar, McCook, and Price 1998; Loffler and Hoey 2018; Straub et al. 2022), this rapid loss of biomass would cause a significant decrease in their functioning, reducing the surface area available for use as a habitat or food. Therefore, further investigation is needed to determine the interactive effects of ALAN and warming on this species, including under more natural conditions or longer experiments.

While laboratory/mesocosm experiments such as done here are important to unravel potential mechanisms of impacts, results may differ from natural environments, where other factors, such as wave action and turbidity, may alter seaweed responses to ALAN and warming. Therefore, further in situ experiments are needed to provide further information on the effects of ALAN and warming on seaweeds. Future studies could also investigate how seaweeds respond to lower levels of ALAN and whether seaweeds are affected differently by the different spectra of ALAN. Additionally, light intensity was measured in lux, which is the standard unit used in most studies on ALAN, as well as for management decisions (Commonwealth of Australia 2020). However, future studies on the effects of ALAN on seaweeds could also measure photosynthetically active radiation (PAR), which provides specific information on the light available for photosynthesis.

Our results indicate that, independently, ALAN can have positive effects on the overall growth and productivity of the habitat‐forming seaweed *Ecklonia radiata*. However, when warming is applied alongside ALAN, we found an antagonistic effect, highlighting the potential future impacts of multiple stressors in urbanised areas. As ALAN and warming continue to increase in intensity and prevalence worldwide, these findings can help us to predict the combined and independent effects of these stressors on shallow coastal ecosystems.

## Author Contributions

**Amelia Caley:** conceptualization (equal), data curation (lead), formal analysis (lead), investigation (equal), methodology (equal), project administration (lead), visualization (lead), writing – original draft (lead), writing – review and editing (equal). **Ezequiel M. Marzinelli:** conceptualization (equal), investigation (equal), methodology (equal), supervision (equal), writing – review and editing (equal). **Maria Byrne:** conceptualization (equal), investigation (equal), supervision (equal), writing – review and editing (equal). **Mariana Mayer‐Pinto:** conceptualization (equal), funding acquisition (lead), investigation (equal), methodology (equal), project administration (equal), resources (lead), supervision (lead), writing – review and editing (lead).

## Conflicts of Interest

The authors declare no conflicts of interest.

## Supporting information


Data S1

## Data Availability

The data and code that support the findings of this study are available for reviewers in Dryad digital repository: https://datadryad.org/stash/share/7C9LGi2esmI1EhrcebbSeoaCQ‐0wU_zukXpsNu0AehA.
