# Supplementary material for: Antagonistic Effects of Light Pollution and Warming on Habitat‐Forming Seaweeds
Source: Ecol Evol. 2024 Oct 16;14(10):e70420. doi: 10.1002/ece3.70420 (PMC11483544; doi:10.1002/ece3.70420)
Supplement: Supplementary file 1 — Data S1 [file ECE3-14-e70420-s001.docx]

***Supplementary information for the paper:* Antagonistic effects of light pollution and warming on habitat-forming seaweeds**

This supporting file includes:

- **Supplementary Figures**
  - Figure S1 – Photograph of juvenile *Ecklonia* *radiata* and *Sargassum*, taken after the experiment.
  - Figure S2 – Photograph of experimental setup
  - Figure S3 – Plot of mean daily temperature
  - Figure S4 – Plot of mean photosynthetic yield of tagged *Ecklonia* and *Sargassum* individuals
- **Supplementary Tables**
  - Table S1 – Results of analysis testing difference in total length between treatments at experiment start.
  - Table S2 – Results from analysis of *Ecklonia* and *Sargassum* maximum photosynthetic yield

**Supplementary Figures**


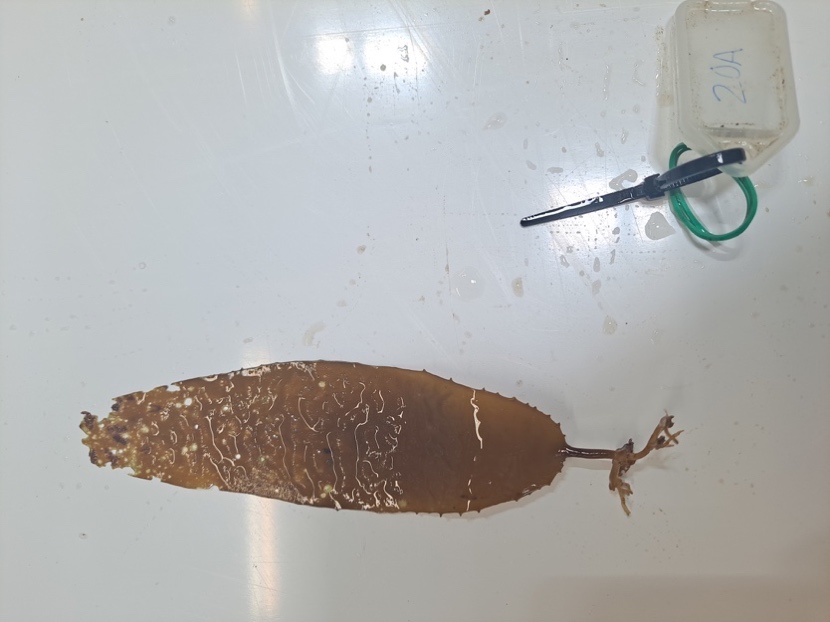

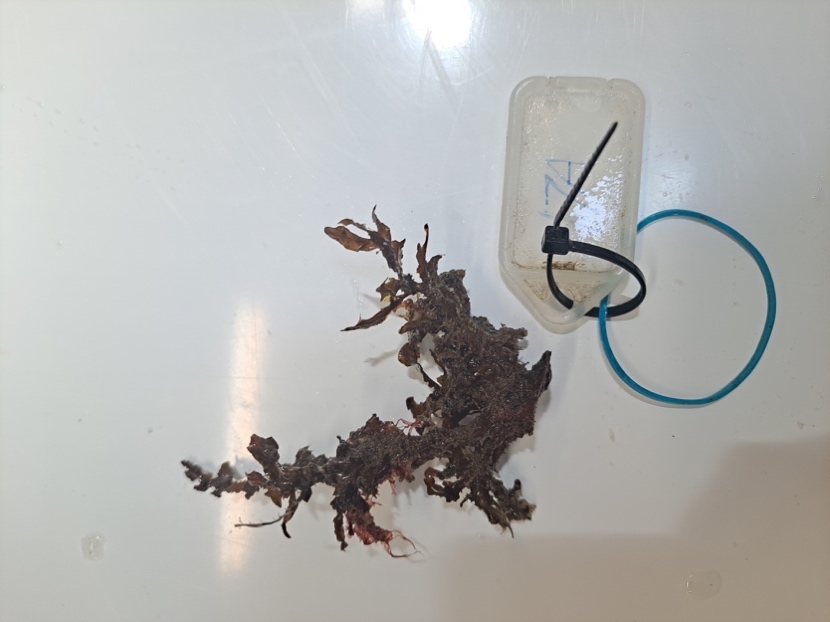


Figure S1. Example of a) *Ecklonia radiata* recruit and b) *Sargassum* juvenile used. These photos were taken after the end of the experiment (~6 weeks post collection), so some bleaching and tissue loss has occurred.


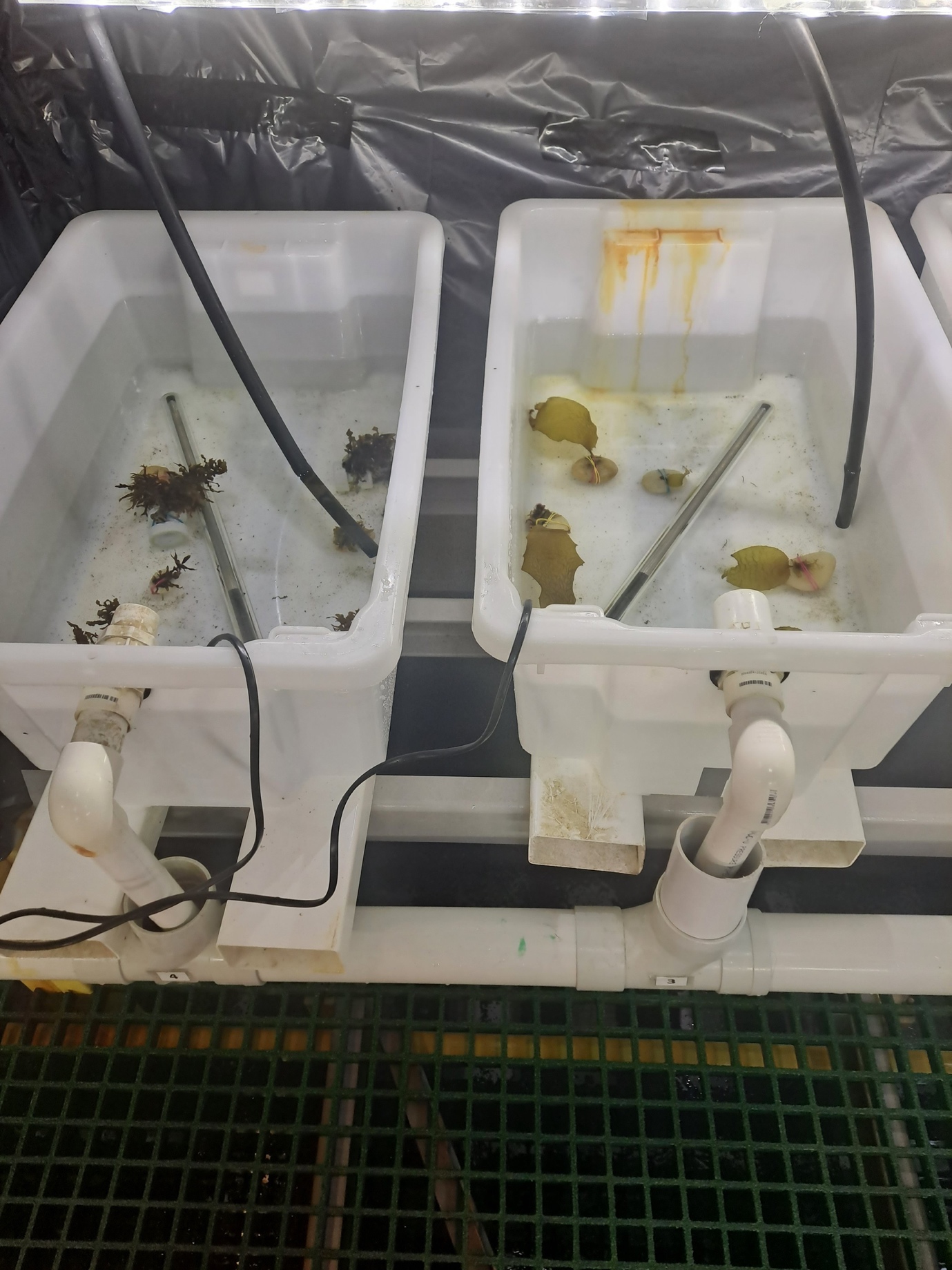


Figure S2. Photograph of Sargassum sp. and Ecklonia juveniles in experiment setup. Photograph was taken during cleaning, so tanks are only partially filled.


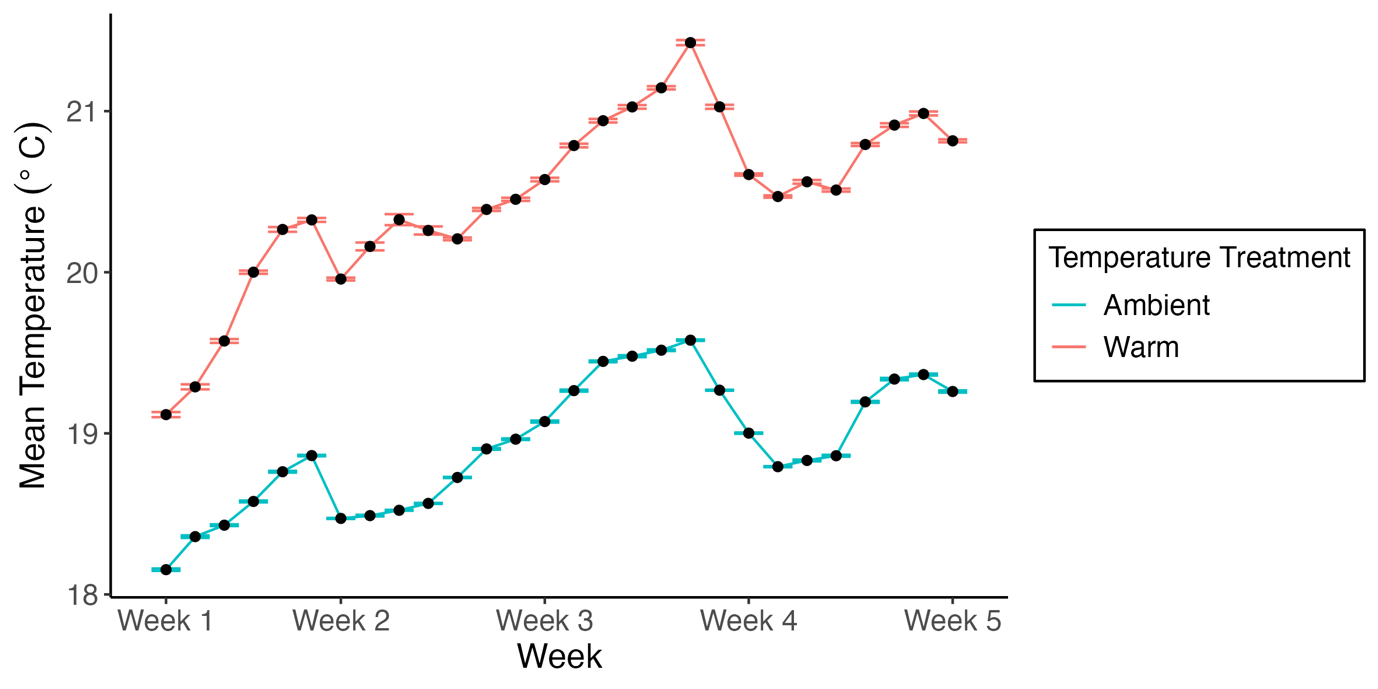


Figure S3. Plot of mean ($\pm$ SE, n = 5 tanks) daily temperature of Ambient and Warm temperature treatments, measured every 30 minutes using HOBO loggers.


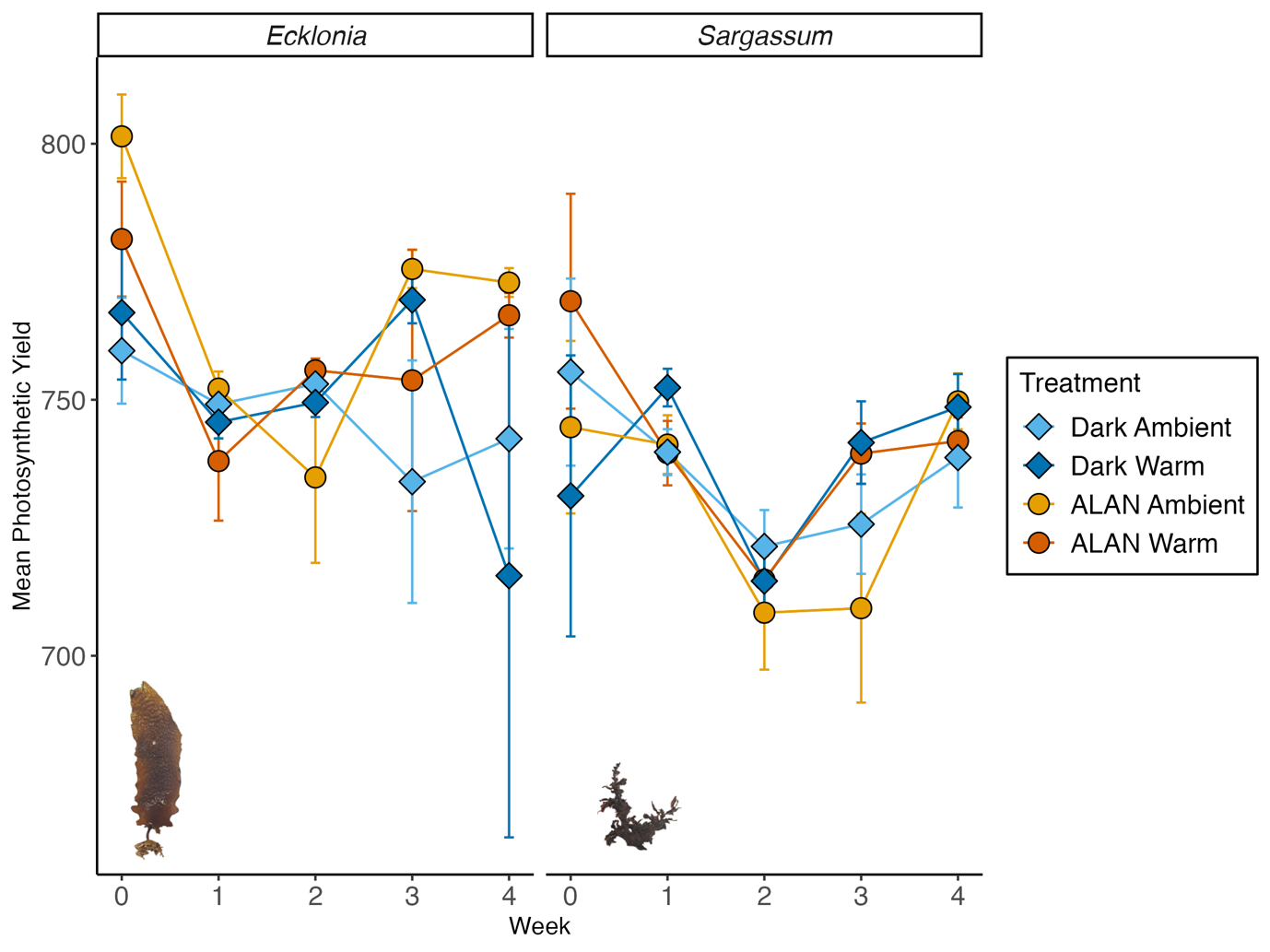


Figure S4. Mean ($\pm$SE, n = 5 tanks) maximum quantum yield of tagged *Ecklonia* and *Sargassum* individuals. ALAN ambient = yellow diamonds, ALAN warm = orange diamonds, dark ambient = light blue circles, and dark warm = dark blue circles

**Supplementary Tables**

Table S1. Results of generalised linear models analysing total length between light and temperature species for a) Ecklonia and b) Sargassum at week 0, showing no significant difference in length between treatments at the start of the experiment

|  | a) *Ecklonia* |  |  | b) *Sargassum* |  |  |
| --- | --- | --- | --- | --- | --- | --- |
|  | Chisq | df | P value | Chisq | df | P value |
| Light | 0.030 | 1 | 0.862 | 0.107 | 1 | 0.744 |
| Warming | 0.338 | 1 | 0.561 | 0.083 | 1 | 0.773 |
| Light $\times$ Warming | 0.288 | 1 | 0.592 | 0.187 | 1 | 0.665 |

Table S2. Analysis of a) *Ecklonia* and b) *Sargassum* maximum photosynthetic yield (Y), using the fixed interacting factors Light (two levels: ALAN, dark) and Temperature (two levels: ambient, dark, with week as a covariate, with individual ID nested in tank as a random factor.

|  | *Ecklonia* yield | | | *Sargassum* yield | | |
| --- | --- | --- | --- | --- | --- | --- |
|  | Chisq | df | P-value | Chisq | df | P-value |
| Light | 3.115 | 1 | 0.078 | 0.082 | 1 | 0.774 |
| Warming | 0.169 | 1 | 0.681 | 0.928 | 1 | 0.335 |
| Week | 4.594 | 1 | 0.032 | 2.689 | 1 | 0.101 |
| Light $\times$Warming | 0.481 | 1 | 0.488 | 0.419 | 1 | 0.517 |
| Light $\times$Week | 0.254 | 1 | 0.614 | 0.578 | 1 | 0.447 |
| Warming $\times$Week | 0.072 | 1 | 0.788 | 0.306 | 1 | 0.580 |
| Light $\times$Warming $\times$Week | 0.089 | 1 | 0.766 | 1.881 | 1 | 0.170 |
